# Risks for Gestational Diabetes Mellitus and Pregnancy-Induced Hypertension Are Increased in Polycystic Ovary Syndrome

**DOI:** 10.1155/2013/182582

**Published:** 2013-11-25

**Authors:** Yunhui Wang, Xiaomiao Zhao, Huidan Zhao, Hong Ding, Jianping Tan, Jingte Chen, Rui Zhang, Ricardo Azziz, Dongzi Yang

**Affiliations:** ^1^Department of Obstetrics and Gynecology, Sun Yat-Sen Memorial Hospital, Sun Yat-Sen University, Guangzhou 510120, China; ^2^The Medical College of Georgia, Augusta, GA 30912, USA

## Abstract

*Objectives*. To evaluate pregnancy outcomes and its determinants in women with polycystic ovary syndrome (PCOS). *Methods*. Two-hundred and twenty pregnant PCOS and 594 healthy women were followed from early pregnancy. Incidences of gestational diabetes mellitus (GDM), pregnancy-induced hypertension (PIH), preterm birth, twinning, and fetal growth restriction (FGR) were determined. *Results*. The incidence of GDM was notably higher among all PCOS combined (54.9%; OR: 2.9, 95% CI: 2.0–4.1) and PCOS subgroups, whether they conceived spontaneously (51.5%; OR: 3.3, 95% CI: 2.0–5.4), or via IVF-ET or ovarian stimulation, compared with controls (14.3%; *P* < 0.001). The incidence of PIH was also higher among all PCOS (10.4%; OR: 2.2, 95% CI: 1.1–4.4) and the subgroup conceiving spontaneously (11.8%; OR: 2.6, 95% CI: 1.1–6.2; *P* < 0.001) but not for those conceiving with IVF-ET (9.1%) or ovarian stimulation (9.4%). Lean women with PCOS (BMI <24 kg/m^2^) had higher incidences of GDM (51.1% versus 14.5%; OR: 5.6, 95% CI: 3.4–9.0) and PIH (8.9% versus 3.2%; OR: 3.0, 95% CI: 1.3–7.1) than lean controls. PCOS woemn with normal glucose tolerance had higher risk for PIH than their comparable control group (OR: 4.0, 95% CI: 1.3–11.7). *Conclusion*. This study suggested that PCOS is an independent risk factor for the development of GDM and PIH. This trial is registered with ChiCTR-RCC-11001824.

## 1. Introduction

Polycystic ovary syndrome (PCOS) is a common reproductive-metabolic disorder of women, associated with ovulatory dysfunction, hyperandrogenism, and polycystic ovaries. It is possible that women with PCOS are at increased risk for complications of pregnancy. Firstly, a high proportion of women with PCOS have an increased risk for metabolic disorders, such as insulin resistance (IR) (50%–70%), impaired fasting glucose (IFG), type 2 diabetes (DM), and obesity [1, 2]. During pregnancy, metabolic dysfunction may manifest as impaired glucose tolerance or gestational diabetes mellitus (GDM) [3, 4], because pregnancy and its associated high progesterone levels may induce a state of enhanced insulin resistance.

These women frequently also use assisted reproductive techniques (ART) to treat anovulation and infertility and demonstrate polycystic ovaries, which increases their risk of multiple gestations. In addition, women with PCOS also have low levels of insulin-like growth factor binding globulin (IGFBP-1), a regulator of IGF-1 activity, which may be associated with fetal growth abnormalities and the development of preeclampsia [5]. These morbidities, individually or combined, may also affect pregnancy outcomes in these women [6].

Although several studies have noted an association between insulin resistance and pregnancy outcomes among women with PCOS and a high prevalence of pregnancy complications, it is still unclear whether adverse pregnancy outcomes are present in PCOS [5–7]. A prior meta-analysis suggested that women with PCOS were at increased risk of developing serious pregnancy complications, such as GDM, pregnancy-induced hypertension (PIH), and preeclampsia; however, this meta-analysis included the effects of fertility treatment and coexistent obesity on the pregnancy outcomes in women with PCOS [8].

More recently, another meta-analysis concluded that significant heterogeneity among the studies reviewed suggested that it is still unclear whether there is a higher risk of GDM in PCOS [6]. In addition, changing diagnostic criteria for GDM and more strict glucose monitoring may affect the incidence of adverse pregnancy outcomes [9, 10]. We therefore conducted the present study to evaluate the incidence of adverse pregnancy outcomes in a cohort of pregnant Chinese patients with PCOS.

## 2. Materials and Methods

### 2.1. Study Population

A total of 220 patients diagnosed with PCOS according to the 2003 Rotterdam consensus criteria [1, 11] were identified prior to and monitored throughout their pregnancy at the Obstetrics Department of the Sun Yat-sen Memorial Hospital (SYSM) of Sun Yat-sen University in China between January 2010 and December 2012. As a control group, 652 pregnant women without PCOS were selected by a computerized random number generator and followed from early pregnancy during the same time period. Pregnancies in PCOS and control groups were confirmed by transvaginal ultrasonography between 6 and 8 gestational weeks.

The exclusion criteria in both groups were as follows: age greater than 40 years, cardiomyopathy accompanied by cardiac insufficiency, active hepatitis, uncontrolled hyperthyroidism, active systemic lupus erythematosus (SLE), serious hematopathy, malignant tumors, serious trauma, smoking, drug/alcohol use, organic pelvic disease, and pregnancy accompanied with acute abdominal disease. Of note, and because the risk of diabetes is known to be increased in PCOS, patients with preexisting diabetes were not excluded in the current study.

### 2.2. Data Collection

The following information was collected prospectively: clinical history, age, body mass index (BMI in kg/m^2^), blood pressure, gestational weight gain, method of conception, length of gestation, singleton or multiple gestation, pregnancy complications, mode of delivery, fetal growth, birth weight, Apgar score, and presence and type of fetal malformations, if any. Patients underwent a 2-hour 75 g oral glucose tolerance test (OGTT) between 24 and 28 weeks of gestation. Subjects were classified as GDM if at least two of the three plasma glucose concentrations obtained equaled or exceeded the following values: fasting glucose 5.1 mmol/L, 1 h level 10.0 mmol/L, and 2 h level 8.5 mmol/L [12].

Other pregnancy complications recorded were (1) PIH, defined as a blood pressure values ≥140/90 mmHg on at least two occasions [8]; (2) preterm delivery (PD), defined as delivery of a fetus with gestational age less than 37 weeks according to the estimated date of delivery and dating based on a mid-trimester ultrasound scan [8]; (3) FGR, defined as fetal growth indices below the 10th percentile for the same gestational age in the Chinese population [8]; (4) large for gestational age (LGA), defined as the birthweight of the neonate equal to or greater than 4 kilogram (kg) [8]; and (5) multiple gestation.

The women diagnosed with GDM were given dietary guidance, and for those with poor blood glucose control with diet therapy alone, insulin therapy was prescribed. All treatments were in accordance with the clinical guidelines for the diagnosis and treatment of diabetes mellitus in pregnancy established in 2007 [13] and the recommendations of the Fourth International Workshop-Conference on Gestational Diabetes Mellitus [14]. The targeted plasma blood glucose range for optimally treated GDM was 3.3–5.3 mmol/L for fasting plasma glucose at night, 4.4–7.8 mmol/L for 1 hour, and 4.4–6.7 mmol/L for 2 hour glucose levels after meal. The health status of the mother and fetus was monitored throughout the pregnancy. Those who had a PD, preeclampsia, antepartum hemorrhage, polyhydramnios, oligohydramnios, fetal distress, fetal serious deformity, fetal death, FGR, or other pregnancy complications were hospitalized for treatment as indicated.

### 2.3. Statistical Methods

The investigators were all physicians trained to perform the data collection for this study. Cases were removed when over 50% of the data were incomplete. (23 cases were not included in the comparison of pregnancy outcomes between PCOS and control group, according to glucose tolerance state, because the information with both OGTT and all the other pregnancy outcomes except glucose tolerance state was not present among them.) Statistical analysis was performed using the Statistical Package for the Social Sciences (SPSS) version 15.0 software package (SPSS, Chicago, IL, USA). Data were expressed as the mean ± SD.

The comparison of mean values between women with PCOS and controls was performed with *t*-tests or rank-sum tests. The incidence of pregnancy complications was compared by chi-square analysis. Subsequently, we stratified analysis of the incidence of pregnancy outcomes according to conception methods (spontaneous conception, IVF-ET, or ovarian stimulation), age at conception (≤30 years or >30 years), BMI (<24 kg/m^2^ (lean) or ≥24 kg/m^2^ (overweight/obesity)) [15], and glucose tolerance state (NGT or GDM), by using chi-square analysis to control for the effects of confounders on the pregnancy outcomes of PCOS. Differences were interpreted as significant at *P* < 0.05.

## 3. Results

Of the 220 women with PCOS, 100 had spontaneous conceptions, 55 conceived with ovulation stimulation, and 65 conceived with in vitro fertilization (IVF). Among PCOS patients, 62 had an early pregnancy loss and 14 had a late pregnancy loss, for a miscarriage of 34.5%. Among the 652 controls selected, 58 subjects suffered a pregnancy loss for miscarriage rate of 8.9%, with an OR of 5.4 (95% CI 4.1, 7.2). In total, 144 women with PCOS and 594 controls with late pregnancy outcomes were analyzed for this study. 

There was no difference in age, BMI, or gravidity between PCOS and control women (30.8 ± 3.9 versus 29.1 ± 3.9 years, 23.0 ± 2.6 versus 20.0 ± 2.4 kg/m^2^, 2.0 ± 1.1 versus 1.7 ± 1.0 pregnancies, resp.), but PCOS had a lower parity than controls (0.8 ± 0.3 versus 0.2 ± 0.4 obstetrical deliveries, resp.). There were no differences between the two groups in terms of race (all were Han race), occupation, socioeconomic condition, or degree education.

Comparing all PCOS combined to control women, PCOS subjects had a higher incidence of twins, GDM, PIH, PD, and FGR than controls, but not a higher incidence of large-for-gestational age (LGA) babies (Table 1). We then compared the pregnancy outcomes between PCOS and controls, subdivided by means of conception (Table 1). The incidence of GDM was higher among PCOS compared to controls, regardless of means of conception. Likewise the incidence of PIH was higher compared to controls among PCOS women conceiving spontaneously, but not among those conceiving using IVF-ET or ovarian stimulation. Women with PCOS who conceived using IVF-ET or ovarian stimulation treatment, but not those conceiving spontaneously, had higher incidences of twins, PD, and FGR.

Since many of the pregnancy complications observed could be due to their higher incidence of multiple gestations, we reanalyzed our data excluding those individuals with multiple gestations. When considered as a whole, PCOS women still had a greater incidence of GDM, PIH, PD, and FGR, but not LGA, compared to controls (Table 2). Considering the PCOS women according to mode of conception, PCOS women who had conceived spontaneously still had a higher incidence of GDM and PIH versus controls, but not a higher incidence of PD or FGR; PCOS women who conceived with IVF-ET or ovarian stimulation demonstrated only increased incidences of GDM or FGR versus controls (Table 2).

We subdivided the women according to BMI (≥24 kg/m^2^ versus <24 kg/m^2^) and age (>30 yrs. versus ≤30 yrs.). Irrespective of BMI or age, pregnant women with PCOS had a higher risk of developing GDM and PD, and of having twins (Table 3) than controls. The incidence of PIH was significantly higher among lean (BMI < 24 kg/m^2^) PCOS and those who were older (age > 30 yrs.); the incidence of FGR was higher among lean PCOS (BMI < 24 kg/m^2^) and those who were younger (age ≤ 30 years).

Finally, we categorized our subjects according to their glucose tolerance state (Table 4). For this analysis we excluded 23 women with PCOS and one control case due to incomplete data. Regardless of glucose tolerance status, pregnant women with PCOS had higher risk of developing PD, FGR, and twins. Women with PCOS and NGT, but not GDM, had a greater risk of developing PIH than their respective controls. The incidence of LGA did not differ between PCOS and control women, for any glucose tolerance subgroup.

## 4. Discussion

This study followed a large cohort of women with PCOS from their first diagnosis of PCOS to the birth of their neonate. It indicated that the women with PCOS were at an increased risk of developing gestational diabetes mellitus, independent of weight and age. Moreover, the lean women with PCOS were also at increased risk of developing pregnancy-induced hypertension, independent of weight, GDM, and ART treatments that might cause twin pregnancy. By considering the means of conception, women with PCOS who underwent IVF-ET or ovarian stimulation treatment had higher risk of having twins, FGR, or preterm delivery, independent of their state of carbohydrate metabolism.

In the current prospective study, the women with PCOS were observed to be at higher risk for developing GDM; however, only the lean women with PCOS had a higher risk of developing GDM than their BMI-matched controls. These findings suggested that PCOS may be a predisposing factor for GDM, independent of obesity [8]. It is important to note that, among the obese population, the effects of PCOS on the presence of GDM were mitigated, while the incidence of GDM among the women with PCOS did not differ significantly from the controls. These results suggested that obesity may also play a role in the development of GDM [16]. The slightly increased incidence of LGA among the women with PCOS who conceived spontaneously might be related to the higher prevalence of GDM among this group [17, 18]. Furthermore, our results indicate that the “typical PCOS” with obesity and the lean phenotype of PCOS are both risk factors for GDM, and they cannot be ignored when managing and following these patients.

Pregnant women with PCOS have a greater risk of pregnancy-induced hypertension compared with controls in the present study, which is in agreement with other studies [19–21]. Moreover, to clarify the effect of PCOS itself on the risk of PIH, the known influencing factors were controlled for the subgroups analysis; these factors included GDM, ART treatments that might result in twin pregnancy [8], BMI, and age. The lean women with PCOS were observed to be at an increased risk of developing PIH, independent of weight, GDM, and ART, which indicated that PCOS itself was an independent risk factor for PIH. Moreover, our study confirmed that the older (age > 30 years) women with PCOS were more susceptible to PIH than the younger women [19].

Androgen excess has also been associated with an increase in carotid Intima-Media Thickness (cIMT) in women with PCOS [22]. Increased cIMT has been widely used as a reflection of preclinical atherosclerotic disease, a contributor to the development of hypertension. Chen et al. [23] reported that hyperandrogenemia in young women with PCOS was associated with hypertension, independent of insulin resistance, age, or obesity. Furthermore, the low-grade chronic inflammation in women with PCOS may also contribute to disorders of inflammatory factors, such as the occurrence of GDM and PIH [20]. Therefore, these factors including hyperandrogenic and chronic inflammatory pathology might contribute to the increased incidence of PIH among PCOS.

Promisingly, using the means of conception to analyze the pregnancy outcomes, there was no increased incidence of twins, FGR, and PD among the women with PCOS who conceived spontaneously compared with the controls. Those who underwent IVF-ET or ovarian stimulation treatments were more likely to have twins, which may cause preterm delivery and FGR [21]. Then, after removal of the cases having twins, the PCOS women with IVF-ET or ovarian stimulation treatments did not have increased risk for PD, but still did for FGR, even in close monitoring during their pregnancy. This finding supplemented those reports that neonates from women with PCOS had lower birth weights than women without PCOS [8, 19, 24], which did not categorize the means of conception. 

There are several advantages of this study. A moderately large cohort of women with PCOS was followed from their first diagnosis of PCOS to their labor and delivery, which is superior to other retrospective studies. Furthermore, the late pregnancy outcomes among the women with PCOS were analyzed by means of conception, BMI, age, and carbohydrate metabolism states to eliminate the confounding effects of these factors on the actual effects of PCOS itself and pregnancy outcomes. 

A major limitation is that this study was not a strict randomized controlled trial (RCT) study and the PCOS women with insulin resistance before pregnancy and all the studied women from the early pregnancy were monitored and intervened, so the pregnancy outcomes of these women did not occur in the nature states, which might introduce bias between the groups. However, it is impossible that we did not manage them since we found the abnormalities. In addition, even in the situation that insulin resistance was treated in the PCOS group, the incidences of the adverse pregnancy outcomes, GDM and PIH, for example, still got higher among them, compared with the control group, independent of the glycometabolism, which makes more sense to some degree. Besides, there was no significant difference of the LGA incidences among all the PCOS subgroups and the controls, neither of the FGR incidences between the PCOS subgroup with spontaneous conception and the controls were observed, although the PCOS group had higher incidence of GDM and PIH and elder age, which illustrated the managements of abnormal glycometabolism and the pregnancy monitoring of the women studies were higher effective. Therefore, on the other hand, our study suggested that, even under close monitoring during pregnancy, the PCOS women were still in higher risks for GDM and PIH. Another limitation of the study is that the PCOS women and the control women were not age and BMI matched. The age and BMI subgroups analysis was used to remedy it. Admittedly, a strict RCT study with age and BMI matched and double-blinded design is needed in the future. 

## 5. Conclusion

In conclusion, the present study indicates that PCOS itself is a risk factor for GDM, independent of weight and age, and for PIH, independent of BMI, age, GDM, and ART treatments.

## Figures and Tables

**Table 1:** Comparison of pregnancy outcome between PCOS and control groups by means of conception.

Group	Twins *n* (%)	GDM *n* (%)	PIH *n* (%)	PD *n* (%)	FGR *n* (%)	LGA *n* (%)
Controls (*n* = 594)	2 (0.3)	85 (14.3)	19 (3.2)	31 (5.2)	9 (1.5)	71 (12.0)
PCOS (*n* = 144)	29^a^ (20.1)	79^a^ (54.9)	15^a^ (10.4)	27^a^ (18.8)	13^a^ (9.0)	17 (11.8)
Spontaneous conception (n = 68)	2 (2.9)	35^a^ (51.5)	8^b^ (11.8)	7 (10.3)	1 (1.5)	10 (14.7)
IVF-ET (n = 44)	17^ad^ (38.6)	20^a^ (45.5)	4 (9.1)	11^af^ (25)	6^af^ (13.6)	5 (11.4)
Ovarian stimulation (n = 32)	10^ad^ (31.3)	16^b^ (50.0)	3 (9.4)	9^af^ (28.1)	6^ae^ (18.8)	2 (6.3)
OR_1_ (95% CI)	52.2 (12.4, 219.7)	2.9 (2.0, 4.1)	2.2 (1.1, 4.4)	2.5 (1.5, 4.4)	0.4 (0.2, 0.7)	1.8 (0.6, 5.2)
OR_2_ (95% CI)	6.0 (0.8, 43.4)	3.2 (2.0, 5.2)	2.6 (1.1, 6.2)	1.4 (0.9, 3.2)	0.1 (0.01, 0.5)	3.4 (1.1, 10.4)

FGR: fetal growth restriction; GDM: gestational diabetes mellitus; LGA: large for gestational age; PD: preterm delivery; PIH: pregnancy-induced hypertension.

^a^ *P* < 0.001, ^b^ *P* < 0.01, ^c^ *P* < 0.05, for all PCOS or PCOS subdivided according to means of conception versus controls.

^d^ *P* < 0.001, ^e^ *P* < 0.01, ^f^ *P* < 0.05, for PCOS conceiving with IVF-ET or ovarian stimulation, versus PCOS conceiving spontaneously.

OR_1_: odds ratio for the comparison of the total PCOS group versus controls.

OR_2_: odds ratio for the comparison of PCOS with spontaneous conception versus Controls.

**Table 2:** Comparison of pregnancy outcomes between PCOS and control groups by means of conception, excluding women with multiple gestation (twins or greater).

Group	GDM *n* (%)	PIH *n* (%)	PD *n* (%)	FGR *n* (%)	LGA *n* (%)
Controls (n = 592)	102 (17.2)	19 (3.2)	30 (5.1)	9 (1.5)	71 (12.0)
PCOS (n = 115)	64^a^ (55.7)	11^b^ (9.6)	13^c^ (11.3)	3^a^ (2.6)	16 (13.9)
Spontaneous conception (n = 66)	40^a^ (60.6)	8^b^ (12.1)	6 (9.1)	0 (0)	10 (15.2)
IVF-ET (n = 27)	13^c^ (48.1)	1 (3.7)	4 (14.8)	1^a^ (3.7)	5 (18.5)
Ovarian stimulation (n = 22)	11^b^ (50.0)	2 (9.1)	3 (13.6)	2^a^ (9.1)	1 (4.5)

For key to abbreviations see Table 1.

^a^ *P* < 0.001, ^b^ *P* < 0.01, ^c^ *P* < 0.05, for all PCOS or PCOS subdivided according to means of conception versus controls.

^d^ *P* < 0.001, ^e^ *P* < 0.01, ^f^ *P* < 0.05, for PCOS conceiving with IVF-ET or ovarian stimulation, versus PCOS conceiving spontaneously.

**Table 3:** Comparison of pregnancy outcomes between PCOS and control group, with the methodology of BMI and age.

Group	GDM *n* (%)	PIH *n* (%)	PD *n* (%)	Twins *n* (%)	LGA *n* (%)	FGR *n* (%)
BMI <24 kg/m^2^						
PCOS (*n* = 90)	46^a^ (51.1)	8^c^ (8.9)	11^cd^ (12.2)	21^a^ (23.3)	13 (14.4)	7^a^ (7.8)
Control (*n* = 566)	82 (14.5)	18 (3.2)	30 (5.3)	2 (0.4)	71 (12.5)	8 (1.4)
OR (95% CI)	5.6 (3.4, 9.0)	3.0 (1.3, 7.1)	2.5 (1.2, 5.2)	85.8 (19.7, 374)	1.2 (0.6, 2.2)	5.9 (2.1, 16.6)
BMI ≥24 kg/m^2^						
PCOS (*n* = 54)	33^c^ (61.1)	7 (13.0)	16^b^ (29.6)	8 (14.8)	4 (7.4)	4 (9.3)
Control (*n* = 28)	9 (32.1)	1 (3.6)	1 (3.6)	0 (0)	2 (7.1)	1 (3.6)
OR (95% CI)	2.9 (1.1, 7.7)	4.0 (0.5, 34.5)	11.4 (1.4, 91.0)		1.0 (0.2, 6.1)	2.2 (0.2, 20.3)
Age ≤30 yrs.						
PCOS (*n* = 69)	32^ae^ (46.4)	4 (5.8)	15^a^ (21.7)	21^ad^ (30.4)	6 (8.7)	7^a^ (10.1)
Control (*n* = 416)	47 (11.3)	13 (3.1)	18 (4.3)	1 (0.2)	47 (11.3)	6 (1.4)
OR (95% CI)	5.7 (3.2, 10.2)	1.9 (0.6, 6.0)	6.1 (2.9, 12.9)	181 (24, 138.0)	0.7 (0.3, 1.8)	7.7 (2.5, 23.7)
Age >30 yrs.						
PCOS (*n* = 75)	46^a^ (61.3)	11^a^ (14.7)	12^c^ (16)	8^a^ (10.7)	11 (14.7)	4 (5.3)
Control (*n* = 178)	37 (20.8)	6 (3.4)	13 (7.3)	1 (0.6)	26 (14.6)	3 (1.7)
OR (95% CI)	6.0 (3.3, 10.9)	4.9 (1.8, 13.9)	2.4 (1.0, 5.6)	21.1 (2.6, 172)	1.0 (0.5, 2.2)	3.3 (0.7, 15.1)

FGR: restriction in fetal growth; GDM: gestational diabetes mellitus; MS: macrosomia; PIH: pregnancy-induced hypertension; PD: preterm delivery.

^a^ *P* < 0.001, ^b^ *P* < 0.01, ^c^ *P* < 0.05, for PCOS versus respective controls.

^d^ *P* < 0.01, ^f^ *P* < 0.05, for PCOS with BMI ≥ 24 kg/m^2^ versus those with BMI < 24 kg/m^2^; or those PCOS with age > 30 yrs. versus age ≤ 30 yrs.

**Table 4:** Comparison of pregnancy outcomes between PCOS and control group, according to glucose tolerance state.

Group	PIH *n* (%)	PD *n* (%)	Twins *n* (%)	LGA *n* (%)	FGR *n* (%)
NGT					
PCOS (*n* = 57)	5^c^ (8.8)	8^b^ (14.0)	11^a^ (19.3)	7 (12.3)	4^c^ (7.0)
Control (*n* = 508)	12 (2.4)	21 (4.1)	1 (0.2)	58 (11.4)	8 (1.6)
OR (95% CI)	4.0 (1.3, 11.7)	3.8 (1.6, 9.0)	121 (15, 960)	1.1 (0.5, 2.5)	4.7 (1.4, 16.2)
GDM					
PCOS (*n* = 64)	9 (14.1)	18^a^ (28.1)	14 (21.9)	7 (10.9)	6^c^ (9.4)
Control (*n* = 85)	6 (7.1)	7 (8.2)	0 (0)	13 (15.3)	1 (1.2)
OR (95% CI)	2.2 (0.7, 6.4)	4.4 (1.7, 11.2)		0.7 (0.3, 1.8)	8.7 (1.0, 74.1)

FGR: restriction in fetal growth; GDM: gestational diabetes mellitus; LGA: large for gestational age; NGT: normal glucose tolerance. PIH: pregnancy-induced hypertension; PD: preterm delivery.

^a^ *P* < 0.001, ^b^ *P* < 0.01, ^ c^ *P* < 0.05, for PCOS and respective controls.
